# Complete Genome Sequence of the Methanococcus maripaludis Type Strain JJ (DSM 2067), a Model for Selenoprotein Synthesis in Archaea

**DOI:** 10.1128/genomeA.00237-18

**Published:** 2018-04-05

**Authors:** Anja Poehlein, Daniel Heym, Vivien Quitzke, Julia Fersch, Rolf Daniel, Michael Rother

**Affiliations:** a Genomic and Applied Microbiology & Göttingen Genomics Laboratory, Institute of Microbiology and Genetics, Georg-August-Universität Göttingen, Göttingen, Germany; b Institute of Microbiology, Technische Universität Dresden, Dresden, Germany

## Abstract

Methanococcus maripaludis type strain JJ (DSM 2067) is an important organism because it serves as a model for primary energy metabolism and hydrogenotrophic methanogenesis and is amenable to genetic manipulation. The complete genome (1.7 Mb) harbors 1,815 predicted protein-encoding genes, including 9 encoding selenoproteins.

## GENOME ANNOUNCEMENT

Methanococcus maripaludis type strain JJ (DSM 2067) was isolated in 1983 from salt marsh sediment (1). This species is an important model organism with respect to its primary energy metabolism, hydrogenotrophic methanogenesis, and amenability to genetic manipulation (2). Furthermore, species of Methanococcus synthesize selenocysteine (sec)-containing proteins (selenoproteins), which are all dispensable in M. maripaludis type strain JJ due to the synthesis of sulfur-containing isoenzymes (3, 4). M. maripaludis JJ is currently the best-developed model organism for studying the mechanism of selenoprotein synthesis in *Archaea*. In this report, we present the complete genome sequence of the type strain.

Genomic DNA of M. maripaludis JJ was isolated using a Wizard Genomic DNA purification kit and assessed using a NanoDrop spectrophotometer and electrophoresis. Extracted DNA was prepared following the protocol for 1D^2^ (SQK-LSK308) sequencing of genomic DNA without any size selection. Sequencing was performed with a MinION device (MinION Mk1B) and SpotON flow cell Mk I (R9.5) using MinKNOWN version 1.10.11 as recommended by the manufacturer (Oxford Nanopore, Oxford, UK). Base calling of the raw data was performed using Albacore version 2.0.1. In addition, isolated DNA was used to generate Illumina shotgun paired-end sequencing libraries, which were sequenced with a MiSeq instrument and the MiSeq reagent kit version 3 as recommended by the manufacturer (Illumina, San Diego, CA, USA). The Unicycler pipeline (5) was used for the combined assembly of the Nanopore and the Illumina reads. The assembly resulted in one circular replicon with a size of 1,714,918 bp and an overall G+C content of 32.92%. Automatic annotation and identification of rRNA and tRNA genes were performed using the software tool Prokka (6). Protein-encoding genes containing selenocysteine (sec) residues were manually identified and annotated. The closed genome contained 10 rRNA genes, whereby two putative rRNA operons were directly adjacent and the third putative operon was located elsewhere on the chromosome. The genome also harbored 36 tRNA genes, 1,259 protein-encoding genes with function prediction, and 556 genes coding hypothetical proteins. Three putative restriction systems were identified. One of these systems is MMJJ_06980, the one which was originally described for this strain (7).

M. maripaludis strain S2 encodes 10 selenoproteins, whereas type strain JJ encodes only 9. Type strain JJ lacks the genes encoding the sec-containing subunit of formylmethanofuran dehydrogenase. Furthermore, in type strain JJ, the genomic organization of the genes encoding the sulfur-containing isoforms of the selenoproteins is more similar to that of (the noncultivated) strain X1 (8) than that of strain S2 (9).

### Accession number(s).

This whole-genome shotgun project has been deposited at DDBJ/EMBL/GenBank under the accession number CP026606.

## References

[1] Jones WJ, Paynter MJB, Gupta R 1983 Characterization of *Methanococcus maripaludis* sp. nov., a new methanogen isolated from salt marsh sediment. Arch Microbiol 135:91–97. doi:10.1007/BF00408015.

[2] Sarmiento BF, Leigh JA, Whitman WB 2011 Genetic systems for hydrogenotrophic methanogens. Methods Enzymol 494:43–73. doi:10.1016/B978-0-12-385112-3.00003-2. 21402209

[3] Stock T, Selzer M, Rother M 2010 *In vivo* requirement of selenophosphate for selenoprotein synthesis in Archaea. Mol Microbiol 75:149–160. doi:10.1111/j.1365-2958.2009.06970.x. 19919669

[4] Stock T, Selzer M, Connery S, Seyhan D, Resch A, Rother M 2011 Disruption and complementation of the selenocysteine biosynthesis pathway reveals a hierarchy of selenoprotein gene expression in the archaeon *Methanococcus maripaludis*. Mol Microbiol 82:734–747. doi:10.1111/j.1365-2958.2011.07850.x. 21992107

[5] Wick RR, Judd LM, Gorrie CL, Holt KE 2017 Unicycler: resolving bacterial genome assemblies from short and long sequencing reads. PLoS Comput Biol 13:e1005595. doi:10.1371/journal.pcbi.1005595. 28594827 PMC5481147

[6] Seemann T 2014 Prokka: rapid prokaryotic genome annotation. Bioinformatics 30:2068–2069. doi:10.1093/bioinformatics/btu153. 24642063

[7] Tumbula DL, Makula RA, Whitman WB 1994 Transformation of *Methanococcus maripaludi*s and identification of a Pst I-like restriction system. FEMS Microbiol Lett 121:309–314. doi:10.1111/j.1574-6968.1994.tb07118.x.

[8] Wang X, Greenfield P, Li D, Hendry P, Volk H, Sutherland TD 2011 Complete genome sequence of a nonculturable *Methanococcus maripaludis* strain extracted in a metagenomic survey of petroleum reservoir fluids. J Bacteriol 193:5595. doi:10.1128/JB.05835-11. 21914896 PMC3187424

[9] Hendrickson EL, Kaul R, Zhou Y, Bovee D, Chapman P, Chung J, Conway de Macario E, Dodsworth JA, Gillett W, Graham DE, Hackett M, Haydock AK, Kang A, Land ML, Levy R, Lie TJ, Major TA, Moore BC, Porat I, Palmeiri A, Rouse G, Saenphimmachak C, Söll D, Van Dien S, Wang T, Whitman WB, Xia Q, Zhang Y, Larimer FW, Olson MV, Leigh JA 2004 Complete genome sequence of the genetically tractable hydrogenotrophic methanogen *Methanococcus maripaludis*. J Bacteriol 186:6956–6969. doi:10.1128/JB.186.20.6956-6969.2004. 15466049 PMC522202

